# Fetal spina bifida in a pregnant woman following omega gastric bypass: Case report and literature review

**DOI:** 10.1016/j.ijscr.2020.04.042

**Published:** 2020-05-11

**Authors:** Lionel El Khoury, Rosa Benvenga, Joel Roussel, Rodolfo Romero, Regis Cohen, Nassir Habib, Jean-Marc Catheline

**Affiliations:** a Department of Digestive Surgery, Centre Hospitalier de Saint-Denis, 2 rue du Docteur Delafontaine, 93200 Saint-Denis, France; b Department of Gynaecology and Obstetrics, Centre Hospitalier Francois Quesnay, 78200 Mantes La Jolie, France

**Keywords:** Bariatric surgery, Omega gastric bypass, Pregnancy, Obesity, Neural tube defects, Congenital malformations

## Abstract

**Introduction:**

Bariatric surgery has been increasingly popular during the last years because of its proven efficacy on obesity and related complications. However, nutrient deficiency is common after surgery, in particular after Roux-en-Y gastric bypass (RYGB) or omega gastric bypass (OGB), due to iatrogenic malabsorption. All vitamins and minerals could be involved, including vitamin B9 which plays an important role in the prevention of neural tube defects during pregnancy. We present a case of a spina bifida in the fetus of a pregnant woman following OGB.

**Case presentation:**

A twenty-six years old young woman underwent OGB five years after weight loss failure post sleeve gastrectomy. Her initial body mass index (BMI) was 42.7 kg/m^2^. Two years after OGB, she became pregnant. On her gynecologist’s advice, the patient discontinued daily vitamin intake before the end of the first trimester, as obstetrical follow-up had been considered appropriate. Regrettably, second trimester ultrasound showed myelomeningocele and surgical abortion was decided during the 25^th^ week of pregnancy.

**Discussion:**

Nutritional status in a pregnant woman is crucial, since it determines the fetal outcome. Biochemical and ultrasound monitoring should be performed regularly, especially in pregnant women with a history of bariatric surgery.

**Conclusion:**

Vitamins, minerals and trace metals deficiencies after bypass bariatric surgery could be prevented by adequate supplementation administered before and during pregnancy.

## Highlights

• Nutritional status in a pregnant woman is crucial. It determines the fetal outcome.
• We added a case of spina bifida after Omega Gastric Bypass.
• Biochemical and ultrasound monitoring should be performed regularly in pregnant women with a history of bariatric surgery.
• Vitamins, minerals and trace metals deficiencies after bypass bariatric surgery could be prevented by adequate supplementation administered before and during pregnancy.

## 1 Introduction

Obesity is reaching very high proportions worldwide and is a major health issue associated to much comorbidity including diabetes mellitus, hypertension, dyslipidemia, sleep apnea syndrome, cardiovascular issues, and many other complications [1]. Moreover, maternal obesity increases risk for pregnancy-related complications including pre-eclampsia, gestational diabetes, hypertension, neonatal death, macrosomia, preterm birth, and congenital abnormalities. It also impacts normal fetal growth [2]. Bariatric surgery is currently the most effective treatment for morbid obesity. Gastric bypass, including Roux-en-Y gastric bypass (RYGB) and omega gastric bypass (OGB), is today the second most common bariatric procedure [3]. RYGB delivers good results regarding weight loss and improvement of obesity-related complications and neonatal outcome [4], but risks of surgical adverse effects including anastomotic stricture, marginal ulcer, fistula, cholelithiasis, bowel obstruction, dumping syndrome and nutritional deficiency remain significant [3]. Thereby, adverse outcome of micronutrient deficit could have significant impact on a new-born infant whose operated mother was noncompliant to medical treatment and vitamin supplementation. After bypass procedures, most of the stomach, duodenum and jejunum, where most of the absorption would take place, are excluded. The overall nutritional intake is decreased due to reduced gastric pouch creation. Both mechanisms cause diminished nutrient and vitamin blood levels [5]. A decrease of 15%–38% of vitamin B9 has been reported [6]. One of the most problematic aspects is birth neural tube defect (NTD) due to vitamin B9 deficiency [7] leading to spinal cord or brain defect, and subsequent development of encephalocele, hydranencephaly, anencephaly and spina bifida including meningocele and myelomeningocele. We present a case of interrupted pregnancy due to spina bifida in a pregnant woman two years after OGB. This work has been reported in line with SCARE criteria [8].

## 2 Case report

A twenty-six years old woman with no known past medical or surgical history, does not use drugs, alcohol, or tobacco, obese as she weighted 125 kg with a BMI of 42.7 kg/m^2^ underwent a SG in June 2010. Her follow-up record stated a 35 kg weight loss after one year. She then progressively gained weight despite dietetic follow-up and five years after SG, her weight was 116 kg and BMI 39.7 kg/m^2^. Multidisciplinary discussion led to perform laparoscopic exploration and a redo-SG [9,10].

Laparoscopy proved impossible due to very numerous severe intra-abdominal adhesions and an open OGB was decided in March 2016. In a first step, a complete viscerolysis was realized. Then, a long and narrow gastric tube was created by applying one horizontal 45 mm roticulator Endo-GIA® stapler (Medtronic®, Minneapolis, MN, USA) at the angle of the lesser curvature, and then 4 vertical 60 mm roticulator Endo-GIA® upward to the angle of His. At 200 cm from the ligament of Treitz, jejunum was mounted in precolic manner, and a side-to-end anastomosis to the gastric tube was performed with a posterior 45 mm Endo-GIA® stapler. Post-operative stage was unremarkable.

The patient was discharged with a vitamin prescription (Multivitamin cocktail including vitamin B9 = 1350 μg daily), as usually delivered in our bariatric surgery department. Six months after OGB her haemoglobin level was 11 g/dl (12.4 g/dl preoperatively). She was then followed up on a regular basis, and her vitamins were normally prescribed. Two years after the OGB, she was still taking her daily vitamins, her weight loss topped at 21 kg, and her weight reached 95 kg with a BMI of 32.5 kg/m^2^. Our patient had adequate serum vitamins levels two years after bypass. She became pregnant in January 2018. Her first trimester ultrasound was reported as normal. During the first trimester of pregnancy, on her gynecologist advice, she stopped taking vitamins. No vitamin assay was carried out. The second trimester ultrasound unfortunately reported a myelomeningocele and surgical abortion was performed at 25 weeks of gestation.

## 3 Discussion

The causes of spina bifida are multifactorial, involving both genetic and environmental factors [11]. Environmental factors include obesity, smoking, antiepileptic drugs such as valproic acid, diabetes, cytogenetic abnormalities [12] and vitamin B9 deficiency [13]. Prevalence of pregnant women who had previously undergone bariatric surgery is increasing along with the obesity pandemic [14]. Regardless of a previous history of bariatric surgery, most nutrient serum levels were found to be lesser in obese than in non-obese pregnant women [15]. This fact could indicate that obese females are more likely to experience complications regarding their future new-borns than thin women. Furthermore, during pregnancy, proper nutritional needs and vitamin requirements increase to ensure proper fetal development. Consequently, a combination of obesity and pregnancy leads to maximal risk of fetal malnutrition and birth defects. This risk is even further increased in patients who have undergone weight loss surgery. Gastric bypass contributes to a malnutritional state because of their iatrogenic effect of both significantly reducing gastric capacity and creating a malabsorptive state since it bypasses a significant part of the small intestines [16]. This phenomenon is yet worsened when patients do not fully comply with the recommended treatment, when follow-up is inadequate or when vitamin supplementation is overlooked. In fact, trouble begins long time before surgery as altered quality of diet and inadequate nutrient intake characterize the obese person [17]. This well known fact is mentioned in numerous publications, including one large prospective study where 24% of obese patients were found to have vitamin B9 deficiency prior to bariatric surgery [18], in addition to other micronutrient deficiencies. It is noteworthy that the presence of decreased vitamin levels preoperatively is the best indicator for post-operative deficiency [19]. This is due to alterations of digestion and absorption, possible vomiting, dumping syndrome, and decreased food intake after gastric bypass whose anatomic alteration implies bypassing most of the stomach, where chlorhydric acid production is essential for folate absorption [20], and all duodenum and proximal jejunal loops, where absorption of most micronutrients, including vitamin B9 occur [21]. Several cases of NTD after RYGB were described in the literature [7,[22], [23], [24]] (Table 1). The prevention of vitamin B9 deficiency can be obtained with simple vitamin oral intake, as retrospective studies indicate normalization of vitamin levels in 80% of patients [25]. Schijns [26] noted less vitamin deficiencies, including vitamin B9, after multivitamin intake in 1160 patients operated on by RYGB. According to the American Society for Metabolic and Bariatric Surgery (ASMBS), a new guideline update published in 2016 [27] defines the nutrient supplementation dietary reference intake (DRI) as 400 μg/day of vitamin B9 for obese females without previous weight loss surgery, with a tolerable level up to 1000 μg in all ages, even for pregnant women. Patients with previous weight loss surgery, including RYGB, should have between 400 and 800 μg daily oral intake and between 800 and 1000 μg if of childbearing age. Levels above 1000 μg daily are not recommended due to possible masking of vitamin B12 deficiency [27]. This is compatible with our recommended prescription at discharge for bariatric surgery patients, since the multivitamin cocktail we use represents an approximate 1350 μg daily intake.

**Table 1:** Fetus with neural tube defect following RYGB and OGB in a pregnant woman (literature review).

	Cases	Procedures	Diseases	Years after surgery	Vitamin B9 intake pre/post-operative
Pelizzo [22]	3	RYGB	Spine dysraphism/Arnold-Chiari	(1,5,18)	No supplement
Moliterno [7]	1	RYGB	Myelomeningocele	1	Non-compliance
Haddow [23]	3	RYGB	Anencephaly, Midthoracic myelomeningocele, Lumbar rachischisis	(2,7,8)	No supplement + alcohol abuse in one case
Martin [24]	3	RYGB	Neural tube defect	4	Non-compliance
Our case	1	OGB	Myelomeningocele	2	Gestational intake stops

RYGB: Roux-en-Y gastric bypass; OGB: Omega gastric bypass.

European guidelines on periconceptional vitamin B9 supplementation [28] show variable recommendations between countries regarding dose and duration of intake. For non-obese patients, recommended vitamin B9 supplementation varies between 400 and 500 μg daily depending on countries, with pregestational durations of between 4 and 12 weeks, and of 8–12 weeks after the beginning of pregnancy. They recommend [28] high doses of vitamin B9 for patients with birth defect risk factors such as obesity, diabetes mellitus, and, epilepsy [28,29]. These doses range from 400 to 5000 μg per day periconceptionally, and in some countries, patients are advised to continue that supplementation throughout the whole pregnancy [29].

According to countries, medical practitioners may be concerned about the potential medicolegal implications in case of non-adherence to these recommendations.

## 4 Conclusion

Nutritional status in a pregnant woman is crucial, since it determines the fetal outcome. Vitamins, minerals and trace metals deficiencies after bypass bariatric surgery could be prevented by adequate supplementation administered before and during pregnancy.

## Funding

We did not have any funding for this study.

## Ethical approval

There was no ethical problem and only a medical abortion has been discussed in muldiscilinary meeting to discuss of ethical abortion.

## Consent

Patient who had abortion gave us her consent.

## Author contribution

All authors participated in the design, data collection of other clinical case, data analysis or interpretation, writing the paper.

## Registration of research studies

Not applicable.

## Guarantor

Régis Cohen is the guarantor of this case report.

## Provenance and peer review

Not commissioned, externally peer-reviewed.

## Declaration of Competing Interest

No potential conflicts of interest include employment, consultancies, stock ownership, honoraria, paid expert testimony, patent applications/registrations, and grants or other funding.
